# Concurrence of idiopathic granulomatous mastitis and breast cancer in a patient on neoadjuvant chemotherapy: A case report

**DOI:** 10.1016/j.ijscr.2024.110702

**Published:** 2024-11-29

**Authors:** Amal Mahmood, Romana Idrees, Lubna Mushtaque Vohra

**Affiliations:** a Medical Student, Bachelor of Medicine and Bachelor of Surgery (MBBS), Aga Khan University, Karachi, Pakistan; b Associate Professor, Department of Pathology and Laboratory Medicine, Aga Khan University Hospital, Karachi, Pakistan; c Associate Professor, Department of Breast Surgery, Aga Khan University Hospital, Karachi, Pakistan

**Keywords:** Idiopathic granulomatous mastitis, Neoadjuvant chemotherapy, Granulomatous lobular mastitis, Case report

## Abstract

**Introduction:**

Idiopathic Granulomatous Mastitis is a rare benign inflammatory disease of the breast.

**Presentation of the case:**

We present a case of 45-year-old woman who was diagnosed with stage II invasive ductal carcinoma in the left breast. While receiving neoadjuvant chemotherapy, she developed idiopathic granulomatous mastitis (IGM) in the left breast after the second cycle. She underwent modified radical mastectomy and has been managed with steroids for IGM on the contralateral side, which developed later in the course of the disease. This is a unique finding with limited literature available on similar cases, to the best of our knowledge.

**Discussion:**

IGM poses a diagnostic challenge requiring histopathology for definitive diagnosis.

**Conclusion:**

Treatment guidelines of IGM are not established making it difficult to manage.

## Highlights

• Idiopathic granulomatous mastitis is common in young parous women.
• Idiopathic granulomatous mastitis is a rare diagnosis concurrent with carcinoma breast.
• Clinical and radiological characteristics of Idiopathic granulomatous mastitis mimics breast malignancy.
• Idiopathic granulomatous mastitis is a very complex disease to treat.

## 1 Introduction

Idiopathic granulomatous lobular mastitis (IGM) is a rare inflammatory breast disease that predominantly affects young parous women aged 20–40. Initially described by Kessler and Wolloch in 1972 [1], numerous cases have been reported since. The annual prevalence of IGM is 2.4 per 100,000 women aged 20–40 years, with higher rates observed among Asian, Hispanic and Arabic descent [2].

IGM poses many diagnostic and therapeutic challenges for the clinicians because it is difficult to distinguish it from malignancy. Both its radiological and clinical presentation closely mimic those of breast carcinoma [3]. The most common presenting complain that has been reported is a painful mass with erythema and inflammation. Other symptoms include nipple retraction, nipple discharge, peau d'orange, edema, and axillary adenopathy. Radiological findings of granulomatous mastitis vary based on the disease's clinical duration [4].It is diagnosed primarily by exclusion with definitive confirmation requiring histopathological examination.

The diagnosis of IGM concurrent with breast carcinoma in the same breast is exceptionally rare, and even more uncommon is the occurrence of IGM in both breasts with malignancy in only one. We aim to discuss a rare case of a 45-year-old female who presented with a lump in her left breast and was subsequently diagnosed with left breast carcinoma. She commenced neoadjuvant chemotherapy and developed IGM in the ipsilateral breast after the second cycle of chemotherapy.

## 2 Case presentation

A 45-year-old housewife, presented to the breast surgery clinic with complain of left breast lump that she had noticed one month prior. There was no pain, discharge, or any other associated symptoms. The patient was pre-menopausal and a known diabetic and hypertensive. She had seven children and breastfed each of them for up to 24 months, with her youngest child being born four years ago. There was no history of breast or ovarian cancer in the family, and the patient denied any addictions. Her past surgical history included a right wrist tetrasomy and cesarean section.

On clinical examination, the patient had firm, ill-defined lump not fixed to the underlying tissue in the upper outer quadrant (UOQ) of the left breast, measuring 2.5 cm × 2 cm. Axillary lymph nodes were not palpable on either side and contralateral breast exam was unremarkable. Mammogram of the affected breast showed a suspicious lesion in the UOQ, classified as BIRADS-4. Ultrasound imaging revealed an irregular hypoechoic solid lesion at 3’o clock position measuring 25.7 × 12.5 × 25. 0 mm. Additionally, the left axillary lymph nodes had a thickened cortex. The right breast displayed focal prominent duct segments with inspissated secretions and a benign looking inflammatory lymph node (BIRADS-2). Ultrasound guided core biopsy with a clip placed within the 3ó clock lesion was performed.

Pathological findings from the core biopsy confirmed the presence of invasive ductal carcinoma of grade 3 in the left breast. A CT scan of the chest, abdomen, pelvis and bones showed no evidence of pulmonary, bony or hepatic metastasis and the clinical stage was determined as T2N1M0. The immunohistochemical profile revealed estrogen receptor (ER) positive, progesterone receptor (PR) negative and Her 2neu 3 + .

**Fig. 1:**
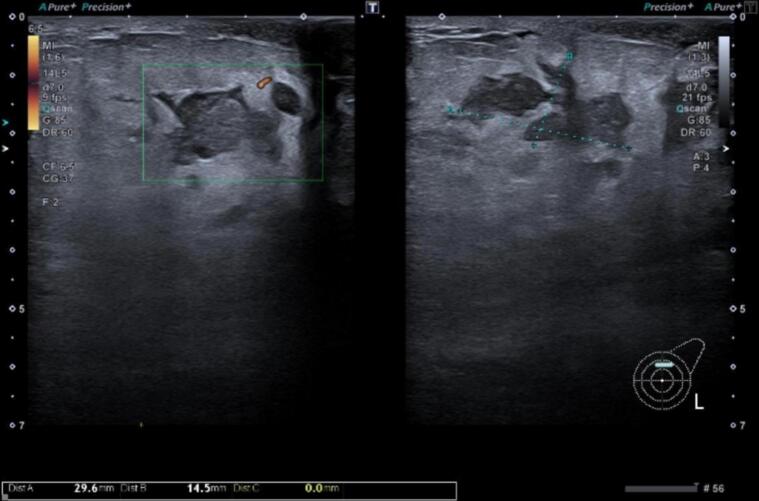
Ultrasound of left breast shows interval development of increase in skin thickness and parenchymal fuzziness with minimal edema and increased echogenicity especially in the *peri* areolar region with tenderness. Interval development of a few new ductal segments with internal non-homogenous avascular echoes.

The patient was started on neoadjuvant chemotherapy (Docetaxel, Carboplatin, Transtuzumab and Pertuzumab) for breast conservation. After the second cycle of chemotherapy, the patient complained of painful breast swelling in the left breast. An ultrasound was performed which revealed a decrease in the size of LUQ lesion to 19 × 14 mm from 25.7 × 25 m but there was an increase in the size of lymph nodes and the internal development of the ductal segments **(**Fig. 1**).** On physical examination, the area was tender and swollen. A repeat CT scan was performed with the intention of ruling out disease progression.

**Fig. 2:**
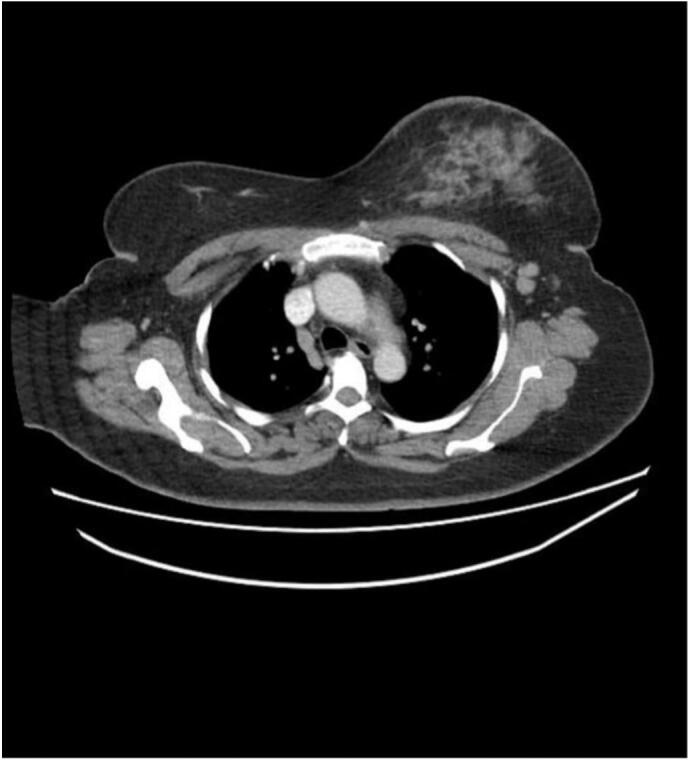
CT scan: interval development of asymmetric enhancement in the upper half of the right breast measuring 34 × 22 mm. Interval increase in the left axillary lymphadenopathy, the largest node measuring 12 mm in short axis.

**Fig. 3:**
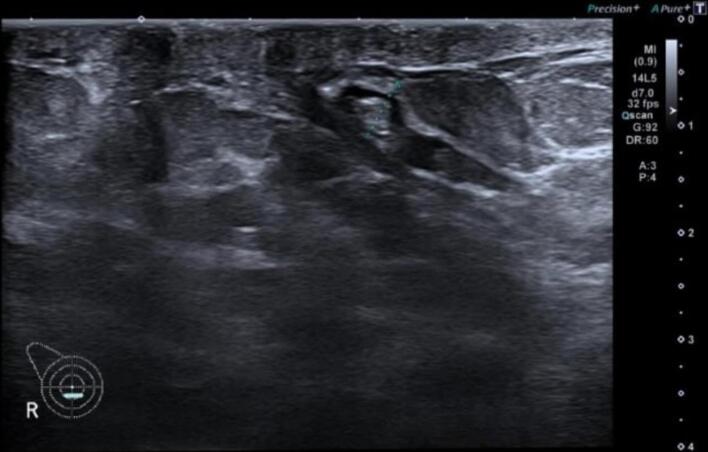
Ultrasound of right breast before treatment shows retro areolar dilated duct with internal avascular echogenic material at 6’o clock.

The CT scan showed ductal development and left axillary lymphadenopathy **(**Fig. 2**)** leading to a revised plan of performing modified radical mastectomy (MRM) under the impression that disease has progressed during neoadjuvant chemotherapy. In the initial phase, the patient was managed with NSAIDS, but she did not respond, and the affected area continued to grow with intense pain. On the day of the procedure, a tender area was noted in the right breast 4 × 5 cm in size which was biopsied during surgery and labeled inflammatory on ultrasound **(**Fig. 3**).**

A modified radical mastectomy of the left breast was performed and a core biopsy was taken from the 12 o'clock position in right breast. Histopathological examination confirmed the diagnosis of idiopathic granulomatous mastitis in the right breast **(**Fig. 5**).** The patient was referred to a rheumatologist for further management. Due to her immunocompromised state, she was initially treated with prednisolone (30 mg).

**Fig. 4:**
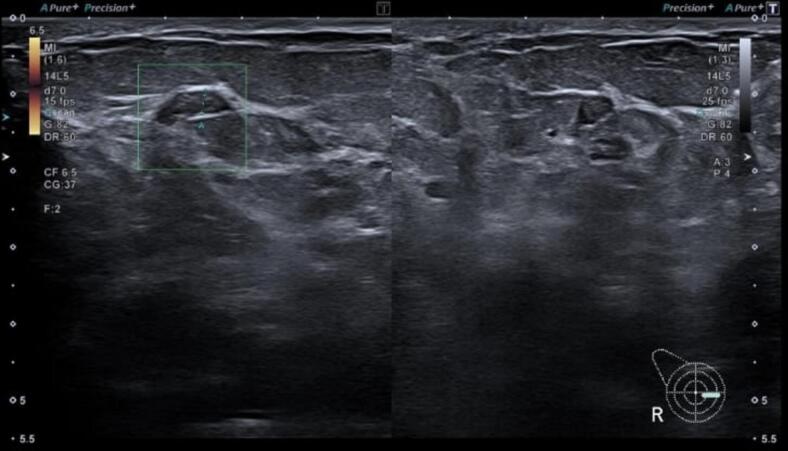
Ultrasound of right breast after conservative treatment showed improvement.

The case was discussed in multidisciplinary team (MTD) meeting, and the decision was made to complete the remaining cycles of THCP chemotherapy, which the patient did, followed by radiotherapy **(**Fig. 4**).** She was having waxing and waning painful area in the right breast with multiple discharging sinuses and was started on methotrexate. She has been doing well since and the disease is now under control.

**Fig. 5:**
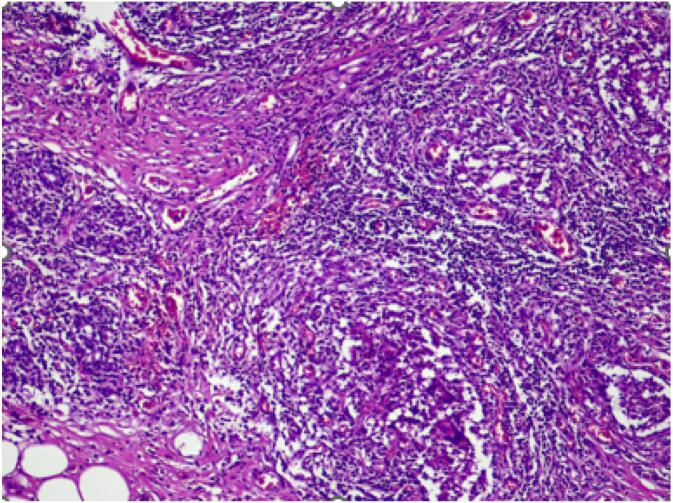
Histological slide showing granuloma formation centered around lobules.

This work has been reported in line with the SCARE criteria [5].

## 3 Discussion

Granulomatous mastitis (GM) is an inflammatory disease of the breast that is characterized by the presence of non-necrotizing granulomatous alterations around the lobules and the ducts of the breast [6]. IGM is usually affects young women of reproductive age and many cases have been reported in women after giving birth or lactation [7].

There are two types of GM: specific and idiopathic. Specific granulomatous mastitis occurs because of an underlying etiological factor or systemic autoimmune conditions whereas Idiopathic granulomatous mastitis (IGM) is a condition in which the cause is unknown, and no etiological factors can be determined [8]. The exact mechanism of IGM development remains unclear, although numerous studies suggest multiple potential etiological factors. These includes the use of oral contraceptive pills, alpha-1 antitrypsin deficiency, hyperprolactinemia, and microbiological agents. Multiple pregnancies and prolonged breastfeeding are also related to an increased of developing granulomatous mastitis [8,9]. Corynebacterium kroppenstedii is the most common microbiological agent detected in IGM cases [10].

The pathogenesis of IGM starts with the damage to the ductal epithelial lining due to ductal ectasia. This is followed by the leakage of ductal contents into the surrounding lobular connective tissue, triggering local inflammation. Lymphocytes and macrophages, then migrate to the periductal areas leading to a local granulomatous response characterized by the formation of noncaseating granuloma [11].

A systematic review of 3090 patients diagnosed with IGM revealed that the most common symptom was a palpable mass affecting 80 % of the patients followed by breast pain in 66 % of the cases [12]. Axillary lymphadenopathy, abscess, erythema and swelling, peau d'orange, and nipple retraction with or without discharge have also been documented. The symptoms of IGM overlap with those of a breast carcinoma creating significant challenges for clinicians in making a diagnosis. This can lead to delays in diagnosing and treating IGM as well as unnecessary mastectomies.

Radiological imaging such as ultrasound, mammogram, and magnetic resonance imaging (MRI) is nonspecific and cannot be used to correctly diagnose IGM. Hence, histopathological examination is needed to make a definitive diagnosis. Compared to fine needle aspiration, ultrasound guided core biopsy is the gold standard for tissue sampling [13]. Histopathological findings in IGM reveal non necrotizing granulomas which are clusters of epithelioid histiocytes with or without giant cells. Giant cells are large cells with multiple nuclei in the same cytoplasm. The affected ducts show loss of acinar structure within the involved breast parenchyma [14].

There are no established treatment guidelines for IGM. Treatment modalities include conservative management and surgical interventions. Steroid therapy with prednisolone at a dosage of 5 to 60 MG per day is recommended for a duration, ranging from one week to 22 months [15]. The use of corticosteroids might lead to the development of Cushing syndrome and recurrence is quite common [16]. In cases where steroid therapy fails, causes side effects, or results in recurrence, immunosuppressants such as methotrexate or azathioprine may be used. In a study by Kim et al. patients were given methotrexate which allowed them to be gradually weaned off the prednisone followed by cessation of methotrexate over 12 months without any evidence of recurrence [15,17]. Our patient experienced right breast pain while on prednisone therapy; thus, methotrexate was considered as the next treatment option.

To the best of our knowledge, this is the only reported case of the development of IGM after neoadjuvant chemotherapy. There have been only a few reported cases of concurrence of IGM with breast malignancy [[18], [19], [20], [21]]. It has not been established whether there is a correlation between IGM and breast carcinoma. A study by Mazlan et al. discussed a rare case of breast carcinoma occurring after chronic IGM [22] illustrating the need to identify the possibility of IGM as a risk factor for the development of breast carcinoma.

IGM has the potential to disrupt and complicate breast cancer treatment strategies. It is a challenging condition, as it closely mimics breast cancer in its presentation, making it difficult to distinguish from a new cancerous lesion through radiological evaluation. Initially, our patient was scheduled for neoadjuvant chemotherapy followed by breast conservation surgery. However, poor chemotherapy response, evidenced by ductal development, edema, and lymphadenopathy with development of IGM in the affected breast necessitated switching to modified radical mastectomy. Future studies should be done to elucidate how IGM affects breast cancer outcomes as understanding this relationship is critical for proper treatment planning and patient prognosis.

## 4 Conclusion

Idiopathic granulomatous lobular mastitis (IGM) is a rare inflammatory breast condition that primarily affects young parous women. It poses significant diagnostic challenges because of its clinical and radiological resemblance to breast carcinoma. A definitive diagnosis requires histopathological examination after excluding malignancy and other conditions. IGM is treated with steroids, immunomodulatory agents or surgery depending upon the clinical scenario and the prognosis is usually favorable.

## Author contribution

Writing- Original Draft: AM

Writing- Review and Editing: AM and LMV

Final approval of the article: AM and LMV

Accountability for all aspects of work: All authors

## Consent

Written informed consent was obtained from the patient for publication of this case report and accompanying images. A copy of the written consent is available for review by the Editor-in-Chief of this journal on request.

## Ethical approval

Not applicable because case reports are exempted from the provision of ethical approval in our institute.

## Guarantor

Dr. Lubna Mushtaque Vohra.

## Research registration number

NA

## Sources of funding

Not applicable.

## Declaration of competing interest

Not applicable.
